# Patient selection in non-operative blunt splenic injury management: A comparative evaluation of MDCT grading systems

**DOI:** 10.6026/973206300222087

**Published:** 2026-04-30

**Authors:** Prashant Vekariya, Pranav H Merchant, Aftab Hossain

**Affiliations:** 1 Department of Radiodiagnosis, Dr. ND Desai Faculty of Medical Science and Research, Dharmsinh Desai University, Nadiad, Gujarat, India; 2 Department of Radiology, Government Medical College, Miraj, Maharashtra, India; 3 Department of Trauma Surgery and Critical Care, All India Institute of Medical Sciences, Jodhpur, Rajasthan, India

**Keywords:** Splenic injury, multidetector computed tomography, vascular injuries

## Abstract

Blunt splenic trauma requires accurate MDCT grading for nonoperative management (NOM) success, yet existing morphology-based systems often fail to account for vascular injuries and hemoperitoneum volume that predict treatment failure. Therefore, it is of interest to evaluate 200 MDCT-scanned patients, comparing grading scales with NOM outcomes, including surgery rates, transfusions, hospital stay, and complications, across injury severities. Higher injury grades, contrast extravasation, large hemoperitoneum, and vascular lesions strongly correlated with NOM failure and adverse events in multivariate analysis. Grading incorporating quantitative hemoperitoneum and vascular markers outperformed traditional morphology-only systems in predictive accuracy for clinical decision-making. Thus, we document integrated MDCT parameters as essential for optimizing NOM selection, thereby reducing unnecessary surgeries and improving risk stratification in splenic trauma care.

## Background:

Blunt splenic injury is one of the most frequently encountered solid organ injuries in abdominal trauma. It remains a major contributor to morbidity in both civilian and road-traffic trauma settings [1]. Historically, splenic injuries were managed surgically due to concerns regarding hemorrhage and delayed rupture. However, with advances in imaging, critical care, and interventional radiology, nonoperative management (NOM) has become the standard of care for hemodynamically stable patients, with reported success rates exceeding 80-90% in appropriately selected cases. The spleen's important immunological role, particularly in protection against encapsulated organisms, has further strengthened the shift toward organ-preserving strategies [2, 3]. The success of NOM depends heavily on early and accurate assessment of injury severity. Multidetector computed tomography (MDCT) has emerged as the cornerstone diagnostic modality for evaluating blunt splenic trauma, owing to its high sensitivity for detecting parenchymal disruption, vascular injury, and hemoperitoneum. MDCT not only confirms the presence of splenic injury but also provides prognostic indicators that guide management decisions, including observation, angioembolization, or surgery [4]. Several grading systems have been developed to stratify splenic injuries based on imaging findings. The most widely used classification is the splenic injury scale proposed by the American Association for the Surgery of Trauma (AAST), which categorizes injuries from grade I to V based primarily on morphological features such as laceration depth and hematoma size [5]. While the AAST grading system has been invaluable in standardizing reporting and research, it was originally developed before the routine use of modern MDCT technology and does not fully account for vascular injuries such as pseudoaneurysm, active contrast extravasation, or splenic devascularisation. These vascular findings have been increasingly recognized as critical predictors of NOM failure [6]. To address these limitations, modified CT-based grading systems have been proposed that incorporate vascular injury patterns and hemoperitoneum volume. The inclusion of these parameters has improved the ability to predict ongoing bleeding, transfusion requirement, and need for intervention [7]. The World Society of Emergency Surgery (WSES) classification, for example, combines hemodynamic status with radiological findings to create a more clinically oriented framework for management. Studies have suggested that systems integrating physiologic status and vascular imaging markers provide better risk stratification than morphology-only grading scales [5, 8]. Despite these developments, there remains variability in how CT findings are interpreted and applied in clinical decision-making. Therefore, it is of interest to compare different MDCT-based splenic injury grading systems in patients with blunt splenic trauma and to evaluate their effectiveness in predicting the success of nonoperative management.

## Materials and Methodology:

## Study design and setting:

This prospective observational study was conducted in the Department of Radiodiagnosis and Trauma Surgery at a tertiary care teaching hospital over 24 months. The study aimed to evaluate and compare different multidetector computed tomography (MDCT) grading systems for blunt splenic injury and determine their role in optimizing patient selection for nonoperative management (NOM).

## Sample size:

A total of 100 patients presenting with blunt abdominal trauma and radiologically confirmed splenic injury were included in the study.

## Inclusion criteria:

[1] Patients aged ≥18 years with blunt abdominal trauma.

[2] Hemodynamically stable patients at admission or stabilized after initial resuscitation.

[3] Splenic injury confirmed on contrast-enhanced MDCT scan.

[4] Patients managed initially with intent for nonoperative treatment.

## Exclusion criteria:

[1] Hemodynamically unstable patients requiring immediate laparotomy.

[2] Penetrating abdominal trauma.

[3] Associated hollow viscus perforation requiring surgery.

[4] Patients with severe traumatic brain injury precluding clinical monitoring.

[5] Previous splenic surgery or known splenic pathology.

## Clinical assessment protocol:

On arrival, all patients underwent:

[1] Primary survey following ATLS guidelines

[2] Hemodynamic evaluation (pulse, blood pressure, shock index)

[3] Laboratory investigations, including hemoglobin, hematocrit, coagulation profile, and serum lactate

[4] Focused Assessment with Sonography for Trauma (FAST)

Patients stabilized after resuscitation underwent contrast-enhanced MDCT.

## Imaging protocol:

All patients were evaluated using contrast-enhanced multidetector CT (64-slice or higher) with arterial, portal venous, and delayed phases.

The following parameters were recorded:

[1] Depth and extent of splenic laceration

[2] Size of sub capsular or intraparenchymal hematoma

[3] Active contrast extravasation

[4] Presence of pseudoaneurysm or arteriovenous fistula

[5] Splenic vascular devascularisation

[6] Volume of hemoperitoneum

[7] Associated abdominal injuries

## Grading systems applied:

Each splenic injury was independently classified according to:

[1] Morphology-based grading using the scale proposed by the American Association for the Surgery of Trauma (AAST)

[2] Combined physiological-radiological grading based on the classification of the World Society of Emergency Surgery (WSES)

[3] Modified CT vascular injury-inclusive grading, incorporating:

1) Active bleeding

2) Pseudoaneurysm

3) Splenic devascularization

4) Hemoperitoneum severity

Radiologists blinded to patient outcomes performed grading independently to reduce observer bias.

## Management protocol:

Patients selected for NOM were managed with:

[1] Strict hemodynamic monitoring

[2] Serial abdominal examination

[3] Hemoglobin monitoring every 6-8 hours initially

[4] Repeat imaging when clinically indicated

[5] Blood transfusion as required

## Angioembolization was performed in patients demonstrating:

[1] Active contrast extravasation

[2] Pseudoaneurysm

[3] Declining hemoglobin despite stability

## Outcome measures:

[1] Success of nonoperative management (no need for surgery during hospital stay)

[2] Need for angioembolization

[3] Blood transfusion requirement

[4] Length of hospital stay

[5] ICU admission

[6] Complications (rebleeding, abscess, delayed rupture)

## Statistical analysis:

Data were entered into SPSS version 25.0.

[1] Continuous variables expressed as mean ± SD

[2] Chi-square test used for categorical comparisons

[3] A p-value <0.05 was considered statistically significant

## Results:

A total of 100 patients with blunt splenic injury were included in the study. All were hemodynamically stable at admission and underwent contrast-enhanced MDCT. Patients were followed for outcomes related to nonoperative management (NOM), the need for angioembolization, and surgical intervention. Overall, NOM was successful in 79 patients (79%), while 21 patients (21%) required intervention (embolization or surgery). Both grading systems demonstrated comparable injury distribution. However, the modified MDCT grading system classified 3% more patients as high risk, reflecting improved identification of vascular involvement. The difference was not statistically significant, indicating that both systems classify anatomical injury similarly (Table 1 - see PDF). Patients with contrast blush had nearly six-fold higher NOM failure (48.4%) compared to those without vascular injury (8.7%). Similarly, high-grade injuries showed failure in 44% of cases, whereas low-grade injuries failed in only 6.8% of cases. Both findings were statistically significant, confirming that vascular injury and high anatomical grade strongly predict need for intervention (Table 2 - see PDF). The modified MDCT grading system demonstrated significantly higher sensitivity (88%) and diagnostic accuracy (85%) compared with the AAST scale (75%). Its higher negative predictive value (95%) indicates superior ability to identify patients suitable for safe nonoperative management (Table 3 - see PDF).

## Discussion:

This prospective study evaluated the effectiveness of MDCT-based grading systems in optimizing patient selection for nonoperative management (NOM) in blunt splenic injury. In our cohort of 200 patients, NOM was attempted in the majority of hemodynamically stable individuals, reflecting current trauma practice trends where conservative treatment is widely accepted as first-line therapy. Contemporary trauma literature reports that nearly 85-90% of splenic injuries are initially managed nono-peratively, with success rates approaching 95% in carefully selected patients. In our study, NOM success decreased progressively with increasing CT grade of injury. Patients with low- grade injuries (Grades I-II) demonstrated success rates above 90%, while moderate injuries (Grade III) showed intermediate success, and high-grade injuries (Grades IV-V) had the highest failure rates. These findings are consistent with large multicenter studies showing failure rates of approximately 2-10% for Grades I-II, 10-20% for Grade III, and up to 40-75% for Grades IV-V injuries when embolization is not performed [9]. Earlier CT-based observational studies also demonstrated a decline in observation success from about 75% in Grade I injuries to nearly negligible success in Grade V injuries [10]. This reinforces the concept that injury grade remains an important but incomplete predictor of outcome. In the present study, a substantial proportion of Grade III-IV injuries were successfully managed conservatively when adjunctive embolization and intensive monitoring were used, suggesting that modern trauma systems allow extension of NOM beyond traditionally accepted limits. A major observation of the present study was that vascular signs on CT- such as contrast extravasation, pseudoaneurysm, or devascularisation-were stronger predictors of NOM failure than morphological grade alone. This aligns with recent CT-based severity index studies, which found that increasing CT severity scores correlated significantly with the risk of delayed vascular complications and treatment failure (p < 0.05). Modern trauma research increasingly emphasizes that CT should not be interpreted solely for laceration depth or hematoma size. Instead, vascular findings and hemoperitoneum volume provide more accurate prognostic information. Observational studies have shown that the inclusion of vascular injury in grading systems improves the prediction of the need for intervention and splenic salvage rates. Our findings similarly demonstrate that modified MDCT classifications incorporating vascular injury provided superior predictive value compared to morphology-based systems alone [11, 12]. In the present study, the availability of splenic artery embolization significantly improved NOM success in high-grade injuries. Patients with vascular injury who underwent early embolization showed higher splenic salvage rates compared with those treated by observation alone. This is consistent with large trauma datasets demonstrating embolization success rates exceeding 90% and a substantial reduction in splenectomy rates over time [13]. Other multicenter experiences have also confirmed embolization as a key factor in extending NOM to higher-grade injuries without increasing mortality [14]. In addition to CT findings, clinical parameters influenced the success of NOM in our cohort. Advanced age, associated injuries, and higher transfusion requirements were significantly associated with treatment failure. Previous trauma analyses have similarly reported that concomitant injuries and physiologic instability increase complication rates and hospitalization duration in patients managed nono-peratively [15]. This highlights the need to integrate clinical variables with radiological grading when selecting patients for NOM [16]. Although MDCT is indispensable in trauma assessment, reliance solely on imaging severity can be misleading. Prior comparative studies have demonstrated discrepancies between CT-predicted and operative injury grades, as well as inter-observer variability in scan interpretation. Our findings support this observation: some patients with moderate CT grades failed NOM due to physiologic instability, while others with severe morphological injury were successfully treated conservatively. Thus, CT grading systems should be viewed as decision-support tools rather than absolute determinants of management. The optimal approach integrates CT findings, hemodynamic status, institutional resources, and the availability of interventional radiology.

## Conclusion:

Data shows that MDCT grading systems play a crucial role in optimizing patient selection for nonoperative management of blunt splenic injury. While traditional morphology-based grading correlates with outcome, systems that incorporate vascular injury patterns and hemoperitoneum provide superior predictive accuracy. The integration of MDCT findings with clinical parameters and early angioembolization significantly improves splenic salvage rates and expands the safe application of conservative treatment.
